# Reply to Hungerford, S.; Bart, N. Blood-Pressure-Monitoring Smartphone Applications: Ushering in a New Era of Wearable Cardiac Devices? Comment on “Vischer et al. Comparability of a Blood-Pressure-Monitoring Smartphone Application with Conventional Measurements—A Pilot Study. *Diagnostics* 2022, *12*, 749”

**DOI:** 10.3390/diagnostics13020291

**Published:** 2023-01-12

**Authors:** Annina S. Vischer, Jana Rosania, Thenral Socrates, Thilo Burkard

**Affiliations:** 1 Medical Outpatient Department and Hypertension Clinic, ESH Hypertension Centre of Excellence, University Hospital Basel, 4031 Basel, Switzerland; 2 Faculty of Medicine, University of Basel, 4056 Basel, Switzerland; 3 Department of Cardiology, University Hospital Basel, 4031 Basel, Switzerland

We would like to thank Drs. Hungerford and Bart for their kind comment [1] on our recent article describing a novel smartphone application attempting to estimate blood pressure [2].

We absolutely agree with Drs. Hungerford and Bart on the growing challenge caused by the increasing prevalence of arterial hypertension worldwide [3]. This necessitates for an improvement of diagnostic possibilities and availability, in order to enable health care systems worldwide to deal with this immense burden [4]. Cuff-based blood pressure measurement devices are currently the recommended technology, as they have proven to safely and correctly measure blood pressure in the case of a recommended validation, and all evidence on morbidity, mortality, and the benefits of the treatment has been assessed using this method [5]. However, they have major disadvantages: (i.) their intermittent nature of measurements; (ii.) discomfort, anxiety, and intrusion of daily activities caused by the inflations; (iii.) the errors achieved through their imperfect shape and position of the cuff; and (iv.) the limited availability of the devices [5]. Nevertheless, we have to keep in mind that nearly all the studies generating the current evidence have had these limitations. In contrast, the wide availability of smartphones lets us believe that this technology can help improve awareness and thus, encourage the better detection of arterial hypertension and may help to develop new markers such as blood pressure variation or help analyze and correlate symptoms such as dizziness, orthostatic symptoms, or presyncope [6,7]. To date, there is no data describing the comparability of continuously measured blood pressure values with our usual blood pressure values and its predicted risk [7].

The unrestricted availability of blood pressure measurement devices comes with a certain risk. If the number, i.e., the blood pressure reading produced by the device, is perceived by the patient and the health care worker as a correct value, but is in fact incorrect, there is a risk of both under- and over-treatment. The patient (and the health care worker) may believe no further action is needed when values are in the normal range and thus could refrain from further measurements for up to five years, according to the European Guidelines for the management of arterial hypertension [8]. On the other hand, it may be decided based on (falsely) elevated blood pressure values that the patient needs antihypertensive treatment, with a risk of increasing instead of lowering cardiovascular risk as well as a risk of syncope and renal dysfunction [9].

Keeping this in mind, we should always use devices that have been validated according to an international standard, for example, the “Universal Standard for the Validation of Blood Pressure Measuring Devices” (AAMI/ESH/ISO) [10,11]. The majority of blood pressure measurement devices on the market, even cuff-based devices, have not been validated [12]. Importantly, these validation protocols are not intended for use in cuffless devices [10,11]. Therefore, generally speaking, cuffless devices should not be used for the evaluation or management of arterial hypertension in daily clinical practice [5].

Accordingly, we have to stress that while the results of this smartphone application examined in our pilot study are very promising, we are far from a clinical utility [2]. First, this was a pilot study including only 50 participants [2]. More than half of the measurements had to be excluded due to insufficient quality, despite being labelled good quality on the application surface [2]. Regardless of the low number of participants and resulting measurements included, we were only scarcely below the putative failure threshold, should the AAMI/ESH/ISO validation protocol be applicable for such a device, oscillometric blood pressure devices be allowed as the gold standard, and all other participants needed for the completion of this protocol result in optimal comparisons [13]. The result of only a handful more “imperfect” results could lead to a definite failure.

The developers of the RIVA Digital application should be applauded for their decision to submit their application at an early stage to test for comparability with conventional blood pressure measurements. Such actions are crucial to prevent the flooding of the market with unvalidated blood pressure measurement devices, and should be facilitated in any future validation protocols, particularly for cuffless devices. An attempt to approach this for the AAMI/ESH/ISO-protocol has been undertaken by our group [13].

In conclusion, while we are very satisfied about the promising results revealed in our pilot study regarding this smartphone application, we first need an internationally accepted validation protocol for cuffless blood pressure measurement devices. Furthermore, we encourage further improvement of the data assessment and refinement of the data interpretation of such a smartphone application, followed by proper validation, before such an application can be used in clinical practice.
